# High Neonatal Mortality Rates in Rural India: What Options to Explore?

**DOI:** 10.5402/2012/968921

**Published:** 2012-11-18

**Authors:** Ravi Prakash Upadhyay, Palanivel Chinnakali, Oluwakemi Odukoya, Kapil Yadav, Smita Sinha, S. A. Rizwan, Shailaja Daral, Vinoth G. Chellaiyan, Vijay Silan

**Affiliations:** ^1^Department of Community Medicine, Vardhman Mahavir Medical College and Safdarjang Hospital, New Delhi 110049, India; ^2^Department of Community Medicine, Indira Gandhi Medical College and Research Institute, Puducherry 605009, India; ^3^Department of Community Health and Primary Care, College of Medicine, University of Lagos, Lagos 23401, Nigeria; ^4^Centre for Community Medicine, All India Institute of Medical Sciences, New Delhi 110029, India; ^5^School of Public Health, Postgraduate Institute of Medical Education & Research (PGIMER), Chandigarh 160012, India

## Abstract

The neonatal mortality rate in India is amongst the highest in the world and skewed towards rural areas. Nonavailability of trained manpower along with poor healthcare infrastructure is one of the major hurdles in ensuring quality neonatal care. We reviewed case studies and relevant literature from low and middle income countries and documented alternative strategies that have proved to be favourable in improving neonatal health. The authors reiterate the fact that recruiting and retaining trained manpower in rural areas by all means is essential to improve the quality of neonatal care services. Besides this, other strategies such as training of local rural healthcare providers and traditional midwives, promoting home-based newborn care, and creating community awareness and mobilization also hold enough potential to influence the neonatal health positively and efforts should be made to implement them on a larger scale. More research is demanded for innovations such as “m-health” and public-private partnerships as they have been shown to offer potential in terms of improving the standards of care. The above proposed strategy is likely to reduce morbidity among neonatal survivors as well.

## 1. The Scale of the Problem of Neonatal Deaths in India

Globally four million deaths occur every year in the first month of life [1]. Almost all (99%) neonatal deaths arise in low-income and middle-income countries [1, 2]. In India alone, around one million babies die each year before they complete their first month of life, contributing to one-fourth of the global burden [1, 3]. The neonatal mortality rate in India was 32 per 1000 live births in the year 2010, a high rate that has not declined much in the last decade [4, 5]. India's neonatal mortality rate dropped significantly, that is, by 25%, from 69 per 1,000 live births in 1980 to 53 per 1,000 live births in 1990 followed by a 15%, decline from 51 to 44 per 1,000 live births between 1991 and 2000. In recent years the NMR has dropped by 15% that is, from 40 per 1000 live births in 2001 to 34 per 1000 live births in 2009 [4]. Urban-rural differences in neonatal mortality exist with the mortality rates higher by 50% in rural (42.5/1000 live births) compared to urban (28.5/1000 live births) areas, as per the National Family Health Survey (NFHS-3) [6]. The common causes of neonatal deaths in India include infections, birth asphyxia, and prematurity which contribute to 32.8%, 22.3%, and 16.8% of the total neonatal deaths, respectively [7, 8].

India is one of the ten countries, along with China, Democratic Republic of Congo, Pakistan, Nigeria, Bangladesh, Ethiopia, Indonesia, Afghanistan, and Tanzania, that account for more than 65% of all intrapartum related neonatal deaths [9]. Despite the recognition of neonatal survival as a key to child survival, poor progress in neonatal survival in India poses concern regarding attainment of the fourth Millennium Development Goal (MDG) target, that is, to reduce under-5 child mortality by two-thirds by 2015. 

## 2. Healthcare Scenario in Rural India

### 2.1. Rural Health Infrastructure

Despite having a comparatively higher neonatal mortality rate, rural India is tackling with the problem of ill equipped public health facilities. The numbers of existing peripheral health facilities fall short of what has been recommended by the government of India. The healthcare in rural areas has been developed as a three-tier structure based on predetermined population norms. The subcenter is the most peripheral institution and the first contact point between the primary healthcare system and the community. Primary Health Centers (PHCs) comprise the second tier in rural healthcare structure envisaged to provide integrated curative and preventive healthcare to the rural population. Community Health Centers (CHCs) form the uppermost tier and their function is mainly to provide specialized obstetric and child care. 

A situational analysis done by the Neonatal Health Research Initiative (NHRI), IndiaClen from 2007–2009, in 24 centers of the country, suggested that less than 20% of the CHCs/PHCs provide essential newborn care services [10]. Also, the availability of a neonatal resuscitation area was relatively low in CHCs (46%) and PHCs (14%) [10]. As per the district level health survey (DLHS-3), newborn care equipment was available in only 27.9% PHCs [11]. Also, while around 76% of the community health centres had newborn care management facilities, just 35.1% had facilities for managing low birth weight babies [11]. These findings underscore the critical condition of the public health facilities that are meant to cater to the health problems of the newborns in rural India.

### 2.2. Status of Trained Healthcare Personnel

Rural public health facilities across the country are having a difficult time attracting, retaining, and ensuring regular presence of highly trained medical personnel especially the gynecologists and pediatricians that are epochal in ensuring and promoting newborn health. Statistics for 2010 suggest a shortfall of 10.3% for doctors at primary health centers (PHCs) [12]. The condition of 4535 community health centers supposed to provide specialized medical care is even more appalling. As compared to requirements for an existing infrastructure, there was a shortfall of 62.6% of specialists at the CHCs, 55.2% of obstetricians and gynecologists and 69.5% of pediatricians [12]. According to the DLHS—Facility Survey (2003), healthcare facilities with newborn care staff and a medical officer trained in newborn care were 59%, 45.0%, and 34% at district hospital, first referral units (FRUs) and CHCs, respectively [13]. As on March 2010, the overall shortfall in the posts of health worker (female)/auxiliary nurse midwife (ANM) was 8.8% of the total requirement [12]. Similarly, in case of health worker (male), there was a shortfall of 64.1% of the requirement. In case of health assistant (female), the shortfall was 31.9% and that of health assistant (male) was 44% [12]. The lack of qualified child care specialists results in a majority of rural households receiving care for their ill babies from private providers, many of whom are less than fully qualified.

## 3. Key Initiatives to Improve Neonatal Health by the Government of India

The Government of India has launched various initiatives envisaging a high priority action with regard to neonatal health. Under National Rural Health Mission (NRHM), Accredited Social Health Activists (ASHAs) are being deployed and assigned the responsibility to create awareness in the community regarding maternal and child health issues [14]. They are further expected to mobilize the community and help them in accessing healthcare services. A safe motherhood intervention named “Janani Suraksha Yojana (JSY)” has been implemented under the NRHM to increase the institutional delivery rates and provide skilled care at birth for the newborn [15]. Under the Reproductive and Child Health program (RCH-II), the quality and reach of antenatal care is planned to be expanded and home-based newborn care using integrated management of neonatal and childhood illness (IMNCI) protocols is envisaged. 

The IMNCI strategy encompasses a range of interventions to prevent and manage the commonest major childhood and neonatal illnesses that cause death, that is, acute respiratory infections, diarrhoea, measles, malaria, and malnutrition [16]. The IMNCI package is planned to be implemented at the level of household and subcentres (through ANMs) and primary health centres (through medical officers, nurses, and lady health visitors). Till October 2011, it has been implemented in 433 districts across the country [17]. 

Facility-based care of neonates (F-IMNCI) is proposed through strengthening of infrastructure, provision of extra nurses, and skills upgradation of physicians and nurses [18]. The Government, with the help of UNICEF, has started setting up special care newborn units (SCNUs) for managing sick newborns [17, 19, 20]. These units have been established at district hospitals and are expected to have a minimum of 12 to 16 beds manned by 3 physicians, 10 nurses, and 4 support staff. A total of 293 SNCUs have been established till the year 2011 [17]. Further, Newborn Stabilization Units (NBSUs) are being set up in First Referral Units (FRUs) and Community Health Centers (CHCs) and they aim to provide care to sick newborns referred from peripheral health facilities [17]. As of October 2011, 1134 NBSUs have been set up [17]. A total of 8582 New Born Care Corners (NBCCs), which are special corners within the labour room where resuscitation, infection control, and early breast feeding can be commenced, have been set up, as of 2011 [17].

Janani Shishu Suraksha Karyakram (JSSK) was launched on 1 June, 2011 with the aim to promote institutional delivery, eliminate out-of-pocket expenses, and facilitate prompt referral through free transport [21]. A program on basic newborn care and resuscitation, named Navjaat Shishu Suraksha Karyakram (NSSK), is being launched to address important interventions at the time of birth that is, prevention of hypothermia and infections, early initiation of breastfeeding, and basic newborn resuscitation [22]. The objective is to have one person trained in basic newborn care and resuscitation at every delivery. This training is being imparted to medical officers, staff nurses, and ANMs at CHC/FRUs and 24 × 7 PHCs where deliveries are taking place [17]. Provision of Comprehensive Emergency Obstetric and New born Care (CEmONC) Services and Basic Emergency Obstetric and Newborn Care (BEmONC) at various levels has also been given due importance. 

Neonatal health is seemingly one of the priority issues in the agenda of the government which gets reflected in the various programs devised and implemented. The worrisome issue is the fact that improving health systems through facility upgradation and ensuring availability of trained manpower and logistics comprise essential prerequisites for the success of these programs/initiatives. The reluctance of trained manpower, especially doctors, to serve in rural areas has become a major impediment in the government's ability to provide quality health services.

## 4. Immediate Challenges

The main obstacles to improving newborn survival are that many babies are born at home without being attended by skilled personnel, faulty home-based newborn care practices are widespread, lack of awareness among care givers limits care-seeking for neonatal illness and even if that is taken care of, lack of trained health workforce adds to the problem. This deficiency in skilled manpower undermines the initiatives by the government to improve neonatal health. Another set of dilemma exists in bringing the neonates and the health system closer to each other. There are broadly two ways of doing so, either bring the health system closer to the neonate or bring the neonate closer to the health system. Both of these are feasible and hold the promise to yield positive results but the real challenge lies in their reproduction and sustainment at the national level.

## 5. Way Forward

### 5.1. Recruiting and Retaining Doctors in Rural Areas

In order to ensure the availability of trained medical personnel in rural areas, we first need to understand the reasons behind the observed shortage. Recruiting trained doctors by all means is one of the essential components towards providing quality maternal and neonatal care services. A recent report documents that out of the 264 paediatricians (including both postgraduates and diploma holders) that are produced annually in India, only around half of them (i.e., 158) are available for public sector service, a large chunk either emigrate or get attracted towards private sector jobs in urban setups [23]. Similar is the scenario for gynaecologists and obstetricians. The predominant reasons for preference to work in urban areas include adequate infrastructural facilities, high salary, and a decent standard of living [24, 25].

Further, in the recent years, there has been substantial emigration of trained doctors to developed countries, much of it coming from lower and middle income countries [26–28]. Among the developing countries, India is the biggest exporter of trained physicians with India-trained physicians accounting for about 10.9% of British physicians and 4.9% of American physicians [29]. A report of the National Commission on Macroeconomics and Health documented that around 10% of the obstetrician(s) and paediatrician(s) that the country produces eventually emigrate [23]. Although the recipient nations and the physicians that emigrate benefit from this migration, the home country loses its important health potentialities.

There is no clear-cut solution to the problem of lack of doctors in rural setup. Interventions in education and financial incentives along with professional support probably have the potential to ease out the problem, as had been seen in rural Australia where the “GPRIP Continuing Medical Education Grants and Locum Grants” designed to assist rural general practitioners to maintain and increase their skills in areas relevant to rural practice helped in their retention in rural areas [30]. The provision of better financial incentives oriented specifically to doctors working in the rural areas might be crucial to attract and retain more doctors in these areas. In Canada, the distribution of doctors was positively influenced by raising fees in rural and underserved areas and reducing fees in “overserved” areas, but in the Philippines, rural incentives had an unintended negative impact due to the fact that local governments were unable to hire healthcare professionals at the high salary levels specified [31–33]. Thus, the experience with paying direct financial incentives, such as rural allowances, has been variable and usually depends on the affordability of resources but this should not undermine the potential it might offer to increase the influx of doctors in rural areas.

Other key initiatives could include establishing rural doctor networks, mentorship programmes, and giving rural practitioners preference in admissions in specialty programs. Exposure to rural areas as part of the training of medical graduates, so they can understand the working conditions and acquire rural clinical skills, is essential and has the potential to yield positive results. This has been documented in Thailand where a majority of graduates continued in rural practice after completing a compulsory rural residency [34]. To prevent brain drain, international scholar exchange programmes could be thought of as an option besides improving healthcare infrastructure and creating an enabling work environment.

### 5.2. Promoting Healthy Domiciliary Newborn Care Practices through Community Mobilization

Poor domiciliary care practices have often been implicated in causing neonatal illness. Several cultural beliefs and traditions that exist in different communities influence care practices. Certain care practices can be deleterious to the health of the baby like applying ghee/oil on cord, early bathing, avoidance of colostrum feeding (considering it as harmful for the baby) and not practicing exclusive breast feeding. Realizing the presence of such traditions in the community and formulating intensive information, education, and communication (IEC) campaigns to address these is required. 

Further, approaches to improve newborn survival should focus on community mobilization as well. There is a need to develop programs where there is a collective involvement of the communities in order to identify problems and their solutions. Several such programs have been implemented in other parts of the globe and have yielded positive results. One such program was the “Bolivia's Warmi program” where the key highlight was participatory planning at the community level, with an emphasis on women's participation to identify obstetric and perinatal health problems and their potential solutions [38]. As a result of the intervention, neonatal mortality decreased from 120 per 1000 live births to 40 per 1000 live births. In rural Nepal, a cluster randomized trial suggested that women's groups facilitated by a local female community worker could reduce neonatal mortality rates by about 30% [39]. In eastern India, the Ekjut trial (2005–2008) evaluated the impact of community mobilization on birth outcomes in three districts of Jharkhand and Orissa. The intervention led to a 45% reduction in neonatal mortality [40]. These studies offer evidence to encourage community involvement and leverage the community resources to bring about improvements in neonatal health. Programs should be designed to acknowledge and maximize these linkages and resources. Further, there is a need to make an effort to integrate community mobilization with health system strengthening.

### 5.3. Promotion of Home-Based Newborn Care (HBNC)

In a review of the evidence-based, cost-effective interventions for reduction of neonatal mortality, Darmstadt et al. documented that a combination of outreach and home-based newborn care at 90% coverage could avert 18–37% neonatal deaths [41]. Home-based newborn care could be explicated as a family as well as community oriented services that involve community mobilization and the empowerment of care givers to demand quality services for their sick newborns [42]. HBNC mainly aims at reducing the neonatal deaths by preventing or treating morbidities such as infections, asphyxia or hypothermia which largely form the preventable causes of mortality. Moreover, they are the underlying causes of nearly 55% of the neonatal deaths in India and addressing them could drastically cut down on the mortality rate [7]. Further, Bhutta et al. have documented that community-based pneumonia case management can lead to a 27% decrease in all-cause neonatal mortality, which indeed is a very high achievement [43].

The most convincing example was set out by Bang et al. in rural Gadchiroli where female village health workers were selected from the local population and were trained to identify and manage asphyxiated newborns [44]. They were also trained to manage neonatal sepsis by providing parenteral antibiotic treatment to sick neonates. In the three years of intervention, there was a 71% reduction in perinatal mortality and a 62% reduction in neonatal mortality compared with the control area. In another example from Sirur, a periurban area near Pune, Maharashtra, India, forty female village health workers were trained to serve a population of 47,000. The village worker identified high-risk cases that required treatment by herself and the nurse, under the supervision of the field medical officer. She also made 3 home visits: on day 1 or soon after delivery and on days 8 and 29. As a result of the intervention, a decline in the neonatal mortality rate of 25% from 51.9 to 38.8 per 1,000 live births was recorded [45]. Other successful examples include trials of home-based care in North India, Bangladesh, Pakistan, and Nepal [46–49].

In addition to creating awareness among community members and care givers in the family through information, education, and communication (IEC) activities, a prerequisite for implementation of home-based care is the development of simple and easily comprehensible standard management guidelines. Further, it would be a challenging task to upscale the home care newborn package to the most vulnerable states such as Uttar Pradesh, Bihar, Jharkhand, Madhya Pradesh, Orissa, and Rajasthan with a high neonatal mortality rate [6].

### 5.4. Introducing Models of Midwifery Care

In rural India, most of the births (53%) occur at home largely unattended by skilled personnel [11]. The lack of a trained personnel predisposes the newborn to a variety of birth related complications mainly birth asphyxia, birth injuries, and infections. Moreover, most of the neonatal deaths occur in the first week of life with a majority of them dying on the first day of birth, thus reflecting the poor intrapartum care that the mother receives [1, 50, 51]. With the shortage of trained personnel, nonavailability of adequate healthcare facilities, poor connectivity to a health facility, and lack of transport facilities, providing care at home through training of midwives/traditional birth attendants (TBAs) would probably be a better option. They can be a vital link between women and the health system, giving advice, encouraging women to go to the clinic to deliver, and accompanying mothers to provide moral support. 

One such successful case study is from Indonesia [52, 53]. In 2003, nearly half of all newborn deaths in the Cirebon district of Indonesia were due to birth asphyxia. In order to address this situation in Cirebon, Program for Appropriate Technology in Health (PATH) supported by Saving Newborn Lives/Save the Children began for training community midwives (bidan di desas). These midwives were taught a series of initial steps for assessing and managing a newborn's condition, including the use of a locally produced tube and mask resuscitation device that could be used in home birth settings. One year after the training, it was found that newborn deaths due to birth asphyxia dropped by 47 percent in the district, at a cost of only $42 per asphyxia death averted [52].

In Zambia, midwife training programs significantly decreased the seven-day neonatal death rate in community health clinics [54]. The midwives were given training in essential newborn care (ENC) and in neonatal resuscitation. After training, the all-cause, 7-day neonatal mortality rate decreased from 11.5 deaths per 1000 live births to 6.8 deaths per 1000 live births. The perinatal mortality rate decreased from 18.3 deaths per 1000 births to 12.9 deaths per 1000 births [54]. Similar examples providing evidence for up scaling of trained midwives in order to lower down the neonatal mortality can be drawn from Sri Lanka, Thailand, Malaysia, and Pakistan [55–59]. 

### 5.5. Focus on Socioeconomic Development

Infant mortality rates (reflecting neonatal mortality as well) are one of the most important indicators of the differentials in health and socioeconomic condition in a community. A substantial progress in lowering down the high burden of neonatal mortality is unlikely unless ways can be found to enhance the economic wellbeing of the lower socioeconomic groups. A pertinent example is that of Kerala, a southern state of India, where the state's achievement of stabilizing population growth, attaining high levels of literacy, and life expectancy have led to a significant decline in the infant mortality rates [60, 61]. In a study done in rural Haryana to document the determinants of neonatal deaths, it was found that the occurrence of deaths was a multifactorial process with involvement of factors at community level, family level (socioeconomic), and biological level and that the socioeconomic determinants explained a large proportion of neonatal deaths [62].

Further, Rahman et al. in their study in Qatar found that low-cost, community-based interventions, on the background of socioeconomic development, had a stronger impact on neonatal and perinatal survival as compared to high-cost institutional interventions [63]. Similar findings documenting the importance of socioeconomic development in reducing the burden of neonatal deaths have been reported from studies done in Chile, Malaysia, Malawi, and Arab countries [64–68].

### 5.6. Capacity Building through Training of Rural Healthcare Practitioners

Neonatal care in rural India is largely provided by a large number of unqualified healthcare providers [69–71]. They are the early providers of neonatal care and often attract a large number of ill newborns because of their easier access and comparatively cheaper treatment that they offer. There is a wide range of quality of services provided by these doctors and it would be useful to standardize their services by providing support in the form of training and technical support. Though it does not qualify as a paragon solution, but this concept would probably score well, given the limited resources the country has. In alignment with what had been advocated by Yadav et al. “Let best not be the enemy of the good,” it would be beneficial to engage these local healthcare providers and equip them with necessary skills to provide acceptable standards of neonatal care until constraints on the supply of qualified and motivated healthcare providers into the system can be alleviated [72]. They could further be involved in promoting key newborn essential care practices as they are popular and acceptable in the community. 

In China, rural healthcare is provided by village doctors who are trained in preventive and curative medicine of both traditional Chinese and allopathic schools. The skills acquired are regularly upgraded by apprenticeship and in-service courses [73, 74]. Another example is from USA where the shortage of physicians in the 1960s paved the way for the emergence of “physician assistants” who were licensed to practice medicine under the supervision of physicians. They made a considerable contribution by working in rural areas which otherwise would not have received any care at all [75, 76]. Successful examples of providing quality healthcare through involvement of local healthcare practitioners can also be seen in Ghana, Mexico, and Bangladesh [77, 78]. 

### 5.7. Introducing a New Cadre of Healthcare Professional

Providing a degree of “Bachelor of Rural Medicine and Surgery (BRMS)” after three-and-a-half years of training, as opposed to five and-a-half years of training for a usual medical graduate, has recently been discussed as one of the possible options to cater to the need of quality healthcare in rural India [79]. The Government of India, in consultation with the Medical Council of India (MCI), is planning to introduce this course in medical schools proposed to be established at district hospitals. The concept of a new degree course of a comparatively shorter duration is to encourage students from rural areas to take up medicine and subsequently provide services in their respective rural areas. The potential impact of selecting medical students of rural origin has been documented by Rabinowitz et al. in a longitudinal study that evaluated the impact of the Physician Shortage Area Program (PSAP) in the USA [80]. The PSAP combined selective admission criteria with a rurally orientated educational program. On multivariate analysis, rural origin was the single variable most strongly associated with rural practice. Studies done in South Africa, Southern Australia, and Canada have also substantiated that the doctors with rural background have more tendency to work in rural areas [81–83].

Students enrolled in the proposed BRMS course will be taught preclinical as well as clinical subjects with more focus on paediatrics and obstetrics/gynaecology. Further, it is envisaged to impart special training in care of the newborn and vaccination [79]. The BRMS graduates would be allowed to practice only in notified rural areas. Chhattisgarh, a state in central India, has come up with the concept of awarding a degree named “rural medical assistants (RMAs)” [84]. This three-year course was a response to a major crisis in human resources for health that the state faced. Three colleges were inaugurated in 2001 and were situated in rural/tribal districts, but with access to a large government hospital (usually the district hospital) to make it possible for clinical teaching and internship [84]. There has been overwhelming positive response to recruitment of RMAs to the most rural and tribal PHC postings, where previously no trained physician existed. 

Establishing a new healthcare cadre would probably have its share of pros and cons. It will certainly improve health care delivery in rural, remote, and tribal areas by providing qualified practitioners but the training of these rural healthcare practitioners will be a major area of concern. It is doubtful as to how overworked, poorly staffed, ill-equipped district hospitals, which cater to thousands of patients, can become quality training grounds for healthcare practitioners. Ensuring that these graduates would practice only in rural areas and not shift to urban setup would be an issue that needs to be addressed. Further, there is a need to document the difference in the quality of care provided by the new cadre of healthcare professional and MBBS graduates.

### 5.8. Investing in Innovations Such As m-Health

The use of mobile phones to improve the quality of care and enhance efficiency of service delivery within healthcare systems is known as mobile health, or m-Health. WHO defines m-Health as the “provision of health services and information via mobile technologies such as mobile phones and Personal Digital Assistants (PDAs)” [85]. m-Health tools have shown promise in providing greater access to healthcare to populations in developing countries, as well as creating cost efficiencies and improving the capacity of health systems to provide quality healthcare. Studies done in Kenya, Sierra Leone and Zanzibar unleash the immense potentialities this innovative concept holds in addressing a wide variety of healthcare challenges [86–88]. 

As earlier discussed, in rural setup, access to healthcare professionals and medical facilities is limited. This can lead to situations where treatable medical condition can become life threatening. Although much work has not been done in context of m-Health in India, yet efforts are required to be made to implement this in the Indian context based on the initial success in other developing countries. The feasibility does not seem to be highly questionable considering the recent increase in the number of mobile phone users in rural areas. According to the press release by the Telecom Regularity Authority of India (TRAI), the number of telephone subscribers in India increased to 943.49 million at the end of February 2012 [89]. The share of urban subscribers had been 65.59% whereas share of rural subscribers had reached 34.41% [89]. Subscription in rural areas had increased from 320.29 million in January 2012 to 324.68 million in February 2012, an increase of 4.39 million in just one month [89]. Now with the recent initiative by the government to provide a subsidy of 20 percent on bills of less than Rs 300 a month to mobile users in rural India, the increase in the number of mobile users could further be expected [90]. 

Mobile telephone short-message service (SMS) can be used for delivering health behaviour change interventions. This service has wide population reach, can be individually tailored, and allows instant delivery, suggesting potential as a delivery channel for health behavior interventions. Researchers in Korea, Croatia, New Zealand, and United Kingdom have used SMS to deliver information pertaining to diabetes and asthma self-management, smoking cessation, and increasing physical activity and this has proved to be beneficial byincreasing awareness and bringing about the desired behaviour change [91–94]. Mobile technology can also be involved in better training of community health workers in using cellular short messages (SMS) to encode and transmit basic health information such as vital signs and health symptoms to a monitoring computer. Algorithms on the monitoring computer could recognize emergent conditions and send system-generated notification informing the community health worker of the appropriate management of the baby related to the inputted vital signs and symptoms.

### 5.9. Strengthening Public-private Partnerships

Given the volume of neonatal care services that are being sought through the private sector in rural areas, one cannot hope to reduce neonatal mortality through public sector interventions alone [69–71]. Because the private sector does not operate within the restrictive confines of a government bureaucracy, one might utilize their services in varied contexts. The advantage with such a partnership could be the wider coverage and increased service utilization. Also, using strengths and skills of each partner enhances efficiency. Successful examples of improving maternal and neonatal health through public private partnerships have been documented in the literature [95–98]. One such example is of Pampers/UNICEF collaboration to eliminate neonatal tetanus [99]. Through this collaboration, over 300 million tetanus vaccines, protecting over 100 million mothers and their babies in 25 of the world's poorest countries, have been provided. 

In even the poorest countries, the private sector is a major provider of goods, services, and information for maternal and child health. There could be different ways to involve the private sector, depending on the resources available and the need of services. One of the strategies could be to use microfinance to allow private sector doctors and other healthcare providers to provide quality practices. One such innovative scheme in India is the Chiranjeevi Scheme in Gujarat [98, 100]. It is an innovative health financing scheme covered through public-private partnership for emergency obstetric care and emergency transport services, for women belonging to below poverty line (BPL) category. Private gynecologists are contracted for services that involve conducting normal and complicated deliveries. The financial package is worked out based on 100 deliveries.

Basinga et al. have published an evaluation of a pay for performance (P4P) scheme implemented in Rwanda [101]. P4P scheme involves for-profit organizations who are provided incentives based on improvements in utilization and quality of care. Statistically significant improvements were observed in the maternal and neonatal health (MNH) indicators of institutional delivery and quality of prenatal care which increased by 21%, and 7.6%, respectively over baseline in the P4P districts [101, 102]. 

Introducing public-private partnerships to improve the quality of maternal and child health services is not new in India. Key examples include Vande Mataram Scheme in West Bengal which involves private sector for provision of safe motherhood and family planning services, Janani Express Yojna in Madhya Pradesh for transportation in case of obstetric emergencies, and use of vouchers in Uttar Pradesh where reproductive and child health services for below poverty line (BPL) women and children are provided through private practitioners [103, 104]. Under National Rural Health Mission (NRHM), several initiatives based on public-private partnerships have been or/are planned to be implemented. The key issues include sustainability of such initiatives and ensuring that quality services are being provided. 

## 6. Conclusions

To conclude, the neonatal mortality rate in India is still high and skewed towards rural areas. Much of the problem lies in the nonavailability of trained manpower and this in turn influences the quality of care the neonates receive. Bringing qualified health professionals to rural, remote, and underserved areas is a challenging task which needs to be addressed urgently to avert neonatal deaths. Other options such as training of local rural healthcare providers and traditional midwives, promoting home-based newborn care, creating community awareness and community mobilization along with strengthening public-private partnerships should be explored further, as evidence generated from previous studies and large scale projects support these strategies as a way to improve neonatal health. More research should be directed towards upcoming innovations such as m-health in order to exploit the potential they offer in terms of enhancing the quality of care.

## Key Note

While the focus should be on devising strategies to recruit and retain trained manpower in rural areas, alternative strategies such as community mobilization, upscaling of home-based newborn care, imparting training and subsequent involvement of local rural healthcare providers and midwives should be attempted as well. More research is required to reveal the potential that innovations such as m-health, telemedicine, and public-private partnership hold in context to improving the quality of care in rural India.
